# Visualization of mucosal field in HPV positive and negative oropharyngeal squamous cell carcinomas: combined genomic and radiology based 3D model

**DOI:** 10.1038/s41598-019-56429-4

**Published:** 2020-01-08

**Authors:** Eva Orosz, Katalin Gombos, Nerina Petrevszky, David Csonka, Istvan Haber, Balint Kaszas, Arnold Toth, Krisztian Molnar, Krisztina Kalacs, Zalan Piski, Imre Gerlinger, Andras Burian, Szabolcs Bellyei, Istvan Szanyi

**Affiliations:** 1 University of Pécs, Medical School, Department of Otorhinolaryngology, Pécs, Hungary; 2 University of Pécs, Medical School, Department of Laboratory Medicine, Pécs, Hungary; 3 University of Pécs, Faculty of Engineering and Information Technology, Pécs, Hungary; 4 University of Pécs, Medical School, Department of Pathology, Pécs, Hungary; 5 University of Pécs, Medical School, Department of Radiology, Pécs, Hungary; 6 University of Pécs, Medical School, Department of Oncotherapy, Pécs, Hungary

**Keywords:** Genetics research, Genetics research, Molecular medicine, Molecular medicine

## Abstract

The aim of this study was to visualize the tumor propagation and surrounding mucosal field in radiography-based 3D model for advanced stage HNSCC and combine it with HPV genotyping and miRNA expression characterization of the visualized area. 25 patients with T1-3 clinical stage HNSCC were enrolled in mapping biopsy sampling. Biopsy samples were evaluated for HPV positivity and miR-21-5p, miR-143, miR-155, miR-221-5p expression in Digital Droplet PCR system. Significant miRNA expression differences of HPV positive tumor tissue biopsies were found for miR-21-5p, miR-143 and miR-221-5p compared to the HPV negative tumor biopsy series. Peritumoral mucosa showed patchy pattern alterations of miR-21-5p and miR-155 in HPV positive cases, while gradual change of miR-21-5p and miR-221-5p was seen in HPV negative tumors. In our study we found differences of the miRNA expression patterns among the HPV positive and negative tumorous tissues as well as the surrounding mucosal fields. The CT based 3D models of the cancer field and surrounding mucosal surface can be utilized to improve proper preoperative planning. Complex evaluation of HNSCC tissue organization field can elucidate the clinical and molecular differentiation of HPV positive and negative cases, and enhance effective organ saving therapeutic strategies.

## Introduction

In addition to the widely-known etiological factors including various chemical carcinogens and poor oral hygiene, the human papilloma viruses (HPV) are major risk factors in the development of head and neck squamous cell carcinomas (HNSCC)^1^. Oral HPV positivity and the number of viral-induced oropharyngeal tumors have significantly higher frequency in males^2^. In the oropharyngeal region the highest HPV positivity can be detected in the palatine tonsils. The HPV positivity ratio of these tumors can amount to 70% compared to the other sub regions^3^. The mucosal surface of lacunas and crypts constitute a strong conjunction between the epithelial mucosa and the lymphatic tissue underneath and is often revealed in the background of tonsillar predilection of HPV initiated carcinogenesis^4^. Clinical investigations have shown revealed remarkable differences between tumors induced by HPV and chemical carcinogenesis: HPV induced tumors particularly emerge at younger ages, and in many cases, the first clinical sign of the malignancy is the lymph node metastasis. The size of primary tumor is frequently small and thereby, it is difficult to explore in the early stage, however, later it often increases in tumor size compared with those HPV negative tumors in the same stage. HPV positive cancers respond much better to oncological and surgical therapy, thus prognosis is generally, far more favorable^5–8^. Considering the differences from aspects of prognosis, behavior and development, a separate TNM classification was released in 2017 for HPV positive oropharyngeal tumors^9,10^.

## Human Papilloma Virus

HPV belongs to the family of *Papillomaviridae* and the HPV Reference Center has classified more than 120 types (Papillomavirus Episteme – NIH). The viral genome in this family is a circular, double-stranded DNA (deoxyribonucleic acid). Regarding the most conserve sequence of the viral genome, which is the L1 open reading frame (ORF) region, the HPV types belong to seven taxonomical genera (alpha-, beta-, gamma-, mupa- and nupa- eta- and thetapapillomaviridae). Protein products translated from L1 and L2 ORFs constitute the viral capside, while E6 and E7 ORFs encode oncoproteins that modulate the viral transformation process. HPV infects the basal cells of the multilayer epithelium. The modification of the epithelial cell differentiation, through regulation of the cell cycle, results in the generation of the continuous reactivation of DNA replication and leads to the replication of the genome of the HPV virus^11–14^ Cutaneous and mucosal invasive types of the HPV can be distinguished, accordingly. Based on oncological risk groups mucosal types can be assigned according to low, intermediate and high risk HPV. The identification of multiple viral infections is frequently observed and the resulting virulence might be influenced by the different genotypes. However interference between different HPV types and the impact on their virulence are events that have not been demonstrated yet. Due to the fact, that clinical observations about the behavior of the HPV positive oropharyngeal cancer differ so much from HPV negative HNSCC, a new TNM classification was needed to be introduced with a specific staging guideline. This statement is underlined by the The Cancer Genome Atlas (TCGA) publication on comparative molecular characteristics of the HPV positive and negative HNSCCs. The difference between the HPV associated and non-viral groups was clearly confirmed^15^. The TCGA study was not able to determine sufficient number of oncogenic mutation HNSCC that can drive the mechanisms of carcinogenesis; beside of this fact many epigenetical alterations were proved to characterize this cancer type. Linking to this concept, a recent publication of Masuda *et al*. stated that HNSCC is strongly linked to changes in epigenetic regulatory^16^. miRNAs as translator molecules between the environmental exposures and genomic regulation might reflect the carcinogenic process through their expression profile changes.

## Cancer Field

Chemical and viral carcinogens constitute a genetically and epigenetically modified field in the intact cells of the mucosal surface as the first step of carcinogenesis. The expression, “cancer field“ or “tissue organization field” is described in the literature. Later, followed by precursor changes, a primary tumor, and recurrence of a tumor or a second primary tumor can develop within this field. The behavior of the oral cavity, oropharyngeal, hypopharyngeal and laryngeal squamous cell carcinoma can be represented via this model of tumor development^17,18^.

## Micro-RNA

micro RNAs (miRNA) are small, 19–25 mer regulator molecules. Interestingly, miRNAs can behave either as oncogenes (oncomirs) or tumor suppressors according to which target mRNA they regulate^19,20^. The comparison of cancer and healthy cells originating from the same tissue presents differences in the expression patterns of miRNA. The analysis of the miRNA expression profile proved to be an appropriate method towards differentiating between normal and tumorous tissues, and selecting subgroups specifically in relation to tissue regarding several tumorous diseases^21^. Previous reviews have observed a discrepancy of miRNA findings across studies focusing on cancers of the head and neck, and there has been no consensus on specific miRNAs that are associated to HPV positivity and HNSCC.

In our study, we aimed to analyze the miRNA profile of oropharyngeal squamous cell carcinomas through a cancer field model in tumors and surrounding healthy tissues. We collected mapping biopsy series of tissue samples from the tumors and from the peritumoral mucosa 1-2-3 cm away from the macroscopic tumor margin. We also aimed to visualize the mucosal field and the tumor propagation in 3D model based on radiography and to combine it with clinical and genomic results. In our experimental design we selected a set of miRNA targets- miR-21-5p, miR-143, miR-155, miR-221-5p- which satisfy the causality criteria for carcinogenesis in HNSCC. Considering the behavioral differences of HPV positive and negative oropharyngeal tumors, we investigated the miRNA pattern alterations among the two groups with highly quantitative droplet digital PCR technique.

## Materials and Methods

### Sample collection

Tissue samples (10–60 mg) were collected from patients undergoing surgical intervention and treatment at the Department of Otorhinolaryngology, Head and Neck Surgery, Clinical Center, University of Pécs. During our study period between 2017 and 2018, 48 patients were diagnosed and received multimodal therapy for oropharynx, hypopharynx and larynx HNSCCs. From this patient cohort 25 patients were selected for miRNA analysis based upon the presence of stage T2-3 squamous cell carcinoma of the oropharynx. Tumor classification and clinicopathological evaluation were carried out by a pathology expert according to the “8th Edition TNM Classification for Head and Neck Cancer, American Joint Committee on Cancer (AJCC), 2017”. Clinicopathological staging, HPV P16 immunohistochemistry and HPV genotyping were performed in all HNSCC cases. Our sample collection strategy followed a strict scheme towards effectively mapping the biopsy originating from the cancer area. We obtained our first sample from the non-necrotic tumor tissue, and next, we collected peritumoral macroscopically normal tissue samples at 1 cm, 2 cm and 3 cm distance from the edge or perimeter of the tumor. Following collection, tissue samples were snap frozen at −80 °C and stored until processing. The clinical and experimental study protocol described here was performed under the license number 2014/5341 (University of Pécs, regional ethical committee). The methods were carried out in accordance with the relevant guidelines and regulations. Informed consent was obtained from all participants who were enrolled in this study.

### RNA isolation and reverse transcription

Cell free total RNA was isolated from 60 µg fresh frozen tissue samples using the Aurum Total RNA mini kit (Cat. no. 7326820; Bio-Rad, Madison, USA). Isolated RNA quality was checked using the Thermo Scientific NanoDrop^TM^ 2000 (Thermo Fisher Scientific, Grand Island, NY). 5 ng total RNA from each sample was reverse transcribed using the miRCURY LNA Universal RT microRNA PCR Kit (Cat. no. 339340; Qiagen).

### Droplet digital PCR analysis

Regarding quantitative PCR analysis, we procured the Qiagen miRCury LNA miRNA PCR assays (Cat. no. Qiagen, Hilden, Denmark), in accordance with the following miRNA targets: hsa-miR-21-5p, hsa-miR-143, hsa-miR-155 and hsa-miR-221-5p. Master mixes were prepared to include 1 µl of miRCury LNA miRNA assay containing the target-miRNA specific forward and reverse primer pair, 12 µl of QX200 ddPCR EvaGreen Supermix (cat. no. 1864034; Bio-Rad) and 9 µl PCR grade water and 2 µl (~100 pg) of cDNA sample. Application onto the plate was perform using approximately 100 pg/µl template concentration. Regarding the negative controls, we prepared a master mix, in which the template cDNA was substituted with PCR grade water (no template control = NTC and a master mix, in which the template cDNA and primer were both substituted with water (no primer control = NTC + NPC). From each master mix, droplets were generated in the automated droplet generator unit of the QX200 automated ddPCR system using Droplet Generation Oil for EvaGreen (Bio-Rad). PCR amplification was carried out in the QX200 Thermo cycler with the following thermic conditions (enzyme activation 1 cycle: 95 °C for 5 min; amplification 40 cycles {96 °C for 30 s and 58–60 °C -primer dependently-for 1 min}, signal stabilization 1 cycle {4 °C for 5 min and 90 °C for 5 min} and hold on, 4 °C. Droplets were immediately analyzed following the PCR reaction in the QX200 Droplet Reader. Fluorescence data were converted into concentrations according to the Poisson distribution statistical analysis used by the QuantaSoft® Analysis Pro software, version 1.0.596 (Bio-Rad, CA, USA).

### HPV P16 Immunohistochemical staining

At present, HPV association of head and neck SCC is determined using p16 immunhistochemistry, and it is widely considered as an important marker with respect to pathogenesis and prognosis^22^. Furthermore, the recently published the 8th edition of TNM-staging handbook also acknowledged the relevance of differentiating HPV and non-HPV related cases^23^.

Some consider p16^INK4a^, the expression of which is thought to be up-regulated due to pRb degradation induced by high-risk HPV E7 protein, a marker which is able to identify tumors with relevant viral oncogene expression^24,25^.

All HNSCC cases were evaluated based on FFPE tissue samples using the standard H&E staining and p16 immunhistochemistry. Regarding the later model, we used the CINtec p16^INK4a^ Detection System (Ref: 9511, Roche mtm laboratories AG, Sandhofer Strasse, Mannheim, Germany). The immunostaining was performed according to the recommended manufacturer’s protocol.

Additionally, the p16 positive staining was concluded based on the identification of strong nuclear and cytoplasmic staining in at least 70% of malignant cells. No staining, faint nuclear and cytoplasmic staining with background marking, small foci of staining or staining in non-malignant cells were considered negative results. The positive control was a cervical squamous cell carcinoma previously proven to be p16 positive, as part of the standard clinical laboratory procedure.

### DNA extraction and HPV genotyping

60 µg of fresh frozen tumor tissue sample was used for the total DNA extraction using the HighPure template preparation kit (Cat. no. 11796828001; Roche, Molecular Diagnostics, Mannheim, Germany). DNA concentration and quality was examined using NanoDrop spectrophotometry.

Linear array HPV genotyping test (Cat. no. 04391853 190; Roche Molecular Diagnostics) was used to determine specifically, which HPV genotypes were present in the tissue samples. The test detects 37 human papillomavirus genotypes: 6, 11, 16, 18, 26, 31, 33, 35, 39, 40, 42, 45, 51, 52, 53, 54, 55, 56, 58,59, 61, 62, 64, 66, 67, 68, 69, 70, 71, 72, 73 (MM9), 81, 82 (MM4), 83 (MM7), 84 (MM8), IS39, and CP6108^23^.

The Linear Array is a qualitative test which uses biotinylated primers sets PGM09/PGMY11 and PC04/GH20 for simultaneous amplification of a 450 bp and 268 bp fragments of the HPV L1 gene and human beta-globin gene, respectively. Following PCR amplification, genotyping is performed using a single nylon strip coated with HPV type-specific and human beta-globin-specific oligonucleotide probes^26^. Testing was performed in accordance with the recommended manufacturer’s instructions.

### Statistical analysis

Concentration data acquired from QuantaSoft software (BioRad) were next exported and analyzed for quantitative differences between the tissue samples according to histopathology, distance from the tumor, and, the location and HPV status were analyzed using IBM SPSS Statistics 21.0 for ANOVA with statistical significance level p ≤ 0.05.

### Image reconstruction for illustration of sample collection sites

CT DICOM data including arterial and venous phase contrast-enhanced high resolution series (slice thickness = 0.75 mm, matrix = 512 × 512, pixel size = 0.445 mm) of the patient above were converted to nifti format using MRIConvert 2.1.0 (Lewis Center for Neuroimaging, University of Oregon, USA) and loaded in 3DSlicer 4.1^27,28^. Based on contrast enhancement, anatomy and asymmetry, tumor mass was delineated in axial plane slices by a radiologist using 3DSlicer segment editor draw tool. Tumor contours were revised and modified if necessary by a board-certified neuroradiologist with over 10 years of clinical experience.

Using the segment editor module of 3DSlicer 4.1 software 3D segmentation of the tumor was created according to the delineation. Using the same module, the 3D segmentation of the air inside the pharynx was created as well. Using the margin and hollow tools a 1 mm thick boundary was created from the segmentation of the air in order to model the mucous membrane. Using the margin tool on the 3D segmentation of the tumor 10 mm, 20 mm and 30 mm offset volumes were created according to the sample collection site distances. Using a logical operator tool, intersections were created using the segmentations of the mucous membrane and the offset volumes applied to the tumor.

## Results

### miRNA profile analysis associated with HPV positivity

In the study period our clinical site diagnosed 48 patients with HNSCC 34 men and 14 women. After clinicopathological classification of the tumors we enrolled to this study only the patients with stage T2-3 HNSCCs with oropharyngeal location, based on the “8th Edition TNM Classification for Head and Neck Cancer, American Joint Committee on Cancer (AJCC), 2017” (Table 1.)

**Table 1:** Demographical and clinical parameters of the selected tumors.

Characteristics		HPV negative (n = 17)	HPV positive (n = 8)	Total (n = 25)
		N (%)	N (%)	N (%)
Gender	Male	13 (52%)	6 (75%)	19 (76%)
	Female	4 (23.5%)	2 (25%)	6 (24%)
Age	0–45	—	1 (12,5%)	1 (4%)
	45–64	11 (64.7%)	5 (62.5%)	16 (64%)
	65+	6 (24%)	2 (25%)	8 (32%)
Tumor Location	Tonsil	2 (11.8%)	1 (12.5%)	3 (12%)
	Base of tongue	6 (24%)	5 (62.5%)	11 (44%)
	Oropharyngeal wall	2 (11.8%)	—	2 (8%)
	Glossotonsillar sulcus	7 (41.2%)	2 (25%)	9 (36%)
Clinical T stage	1	—	—	—
	2	7 (41.2%)	5 (62.5%)	12 (48%)
	3	10 (58.8%)	3 (37.5%)	13 (52%)
	4	—	—	—
Clinical N stage	0	3 (17.6%)	—	3 (12%)
	1	6 (35.2%)	3 (37.5%)	9 (36%)
	2	8 (47.1%)	5 (62.5%)	13 (52%)
	3	—	—	—
Overall stage	I	—	—	—
	II	3 (17.6%)	4 (50%)	7 (28%)
	III	6 (35.2%)	4 (50%)	10 (40%)
	IV	8 (47.1%)	—	8 (32%)
Risk group	Alcohol	13 (76.5%)	—	13 (52%)
	Smoking	13 (76.5%)	3 (37.5%)	16 (64%)

**Figure 1:**
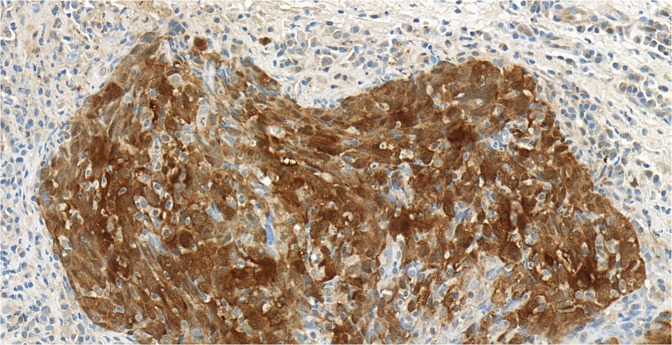
Squamous cell epithelium negative for p16 immunohystochemical staining at 40x magnification. Histological section originates from a grade 2 oropharyngeal wall squamous cell carcinoma.

**Figure 2:**
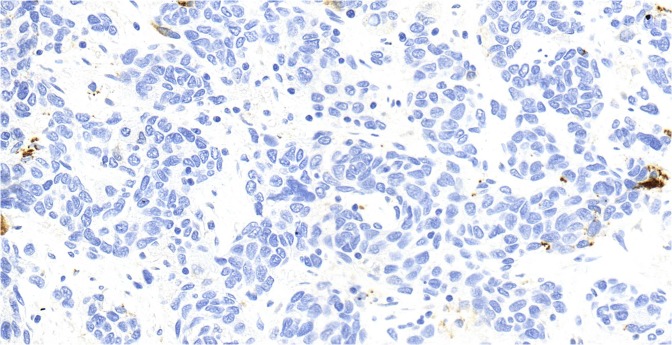
Squamous cell epithelium positive for p16 immunohystochemical staining at 40x magnification. Histological section originates from a grade 3 tonsillar squamous cell carcinoma.

Out of the 25 mesopharyngeal tumors, eight cases proved to be HPV positive (28%) with p16 immunohistochemical staining (Figs. 1,2). Genotyping found seven cases of HPV 16 positivity and one case with combined positivity of HPV 16 and 52 genotypes.

**Figure 3:**
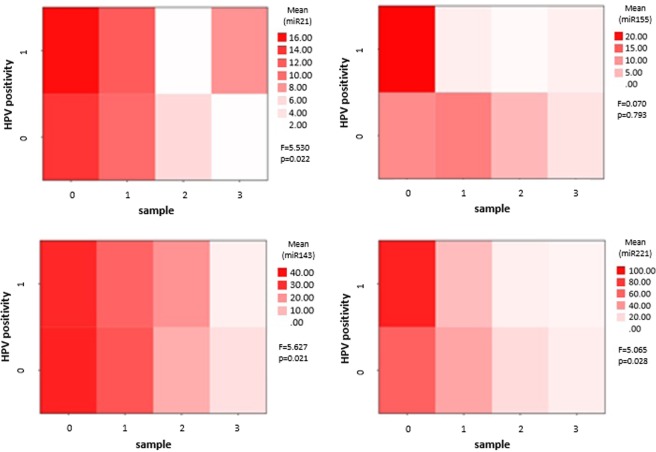
Comparative heat map analysis of HPV positive and negative tumors. Tissue samples are grouped according to HPV positivity and labeled vertically (1 for HPV positive and 0 for HPV negative tissues). The horizontal scale shows the mucosal tissue samples according to distance from the macroscopic tumor margin (0 = tumor, 1 = normal mucosal tissue 1 cm away from the tumor margin, 2 = normal mucosal tissue from 2 cm and 3 = normal mucosal tissue from 3 cm distance from the clinically visible tumor propagation). HPV positive and negative tissue sample series were compared on ANOVA for expression differences of miR-21-5p, miR-143, miR-155 and miR-221-5p. F-probe results and significance levels are shown on the right side of each heat m*a*p. The mean difference was accepted to be significant at the 0.05 level.

HPV positive tumor tissues showed higher expression levels of all investigated miRNA compared to the HPV negative. Significant over expressions in the HPV positive tumor tissue biopsies were found with ANOVA for miR-21-5p (F = 5.53, p = 0.022), miR-143 (F = 5.627 p = 0.021), and miR-221-5p (F = 5.065 p = 0.028). The statistical analysis did not confirm miR-155 to be consequently correlated to HPV infection in the tissue biopsy series (F = 0.70, p = 0,793). Based on our results, we were not able to identity a single miRNA marker specific for HPV positivity, but patterns of the miRNA expression profiles correlated with the presence of HPV (Fig. 3.). miRNA expressions in correlation with the distance from the tumor margin on the peritumoral mucosa showed patchy pattern alterations of miR-21 and miR-155 in HPV positive cases, while gradual change of all investigated miRNAs was seen in HPV negative tumors.

### miRNA profiles correlating with tissue organization field (TOF)

**Figure 4:**
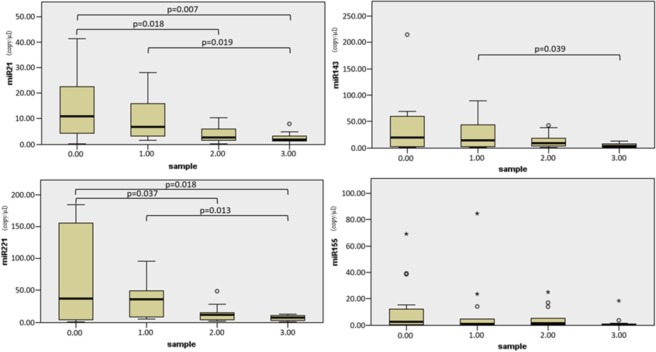
Micro-RNA expression values (y axis: absolute quantification results in copy/μl) according to distance of the biopsy sample from the macroscopic tumor margin (row: 0: tumor, 1: 1 cm from the tumor, 2: 2 cm from the tumor, 3: 3 cm from the tumor). The mean differences were analyzed with ANOVA and significance was maintained at the 0.05 level.

In HPV negative tumors and peritumoral tissues miRNA expression changes followed gradual pattern which depended on the measured distance from the tumor propagation itself. On the peritumoral normal mucosal field, the gradual concentration decrease of two miRNAs (miR-21-5p, miR-221-5p) was found to be statistically significant on the ANOVA test (Fig. 4). miR-143 and -155 also showed a similar miRNA distribution pattern, however, it did not reach the limit of statistical significance.

In the tumors and their neighboring intact mucosa, miR-21-5p showed gradually decreasing values correlating with distance from the tumor propagation. Average miR-21-5p values do not differ significantly among the tumor samples and in the 1 cm peritumoral normal mucosa. We found a significant decrease in miR-21-5p expression levels in tissues at 2 and 3 cm distance from the tumor margin.

The concentration of miR-221-5p was found to be high in tumor tissues while it decreased rapidly in the hystopathologically normal peritumoral mucosa. The standard deviation of the miRNA-221-5p expressions were found to scatter in wider range among the tumor biopsies, while we detected more integrated expression levels in the mucosa biopsies. Mean miR-221-5p expression levels did not significantly differ from the site of the 1 cm peritumoral mucosal region. miR-221-5p levels were equally found to be low in 2 and 3 cm distant biopsies.

Although we noticed gradual down-regulation of miR-143 expression in our cancer field detection model, it was not confirmed to be statistically significant on the ANOVA.

Regarding miR-155, we were not able to detect correlation in the mapping biopsy samples with the tumor propagation field.

#### 3 Dimensional model

**Figure 5:**
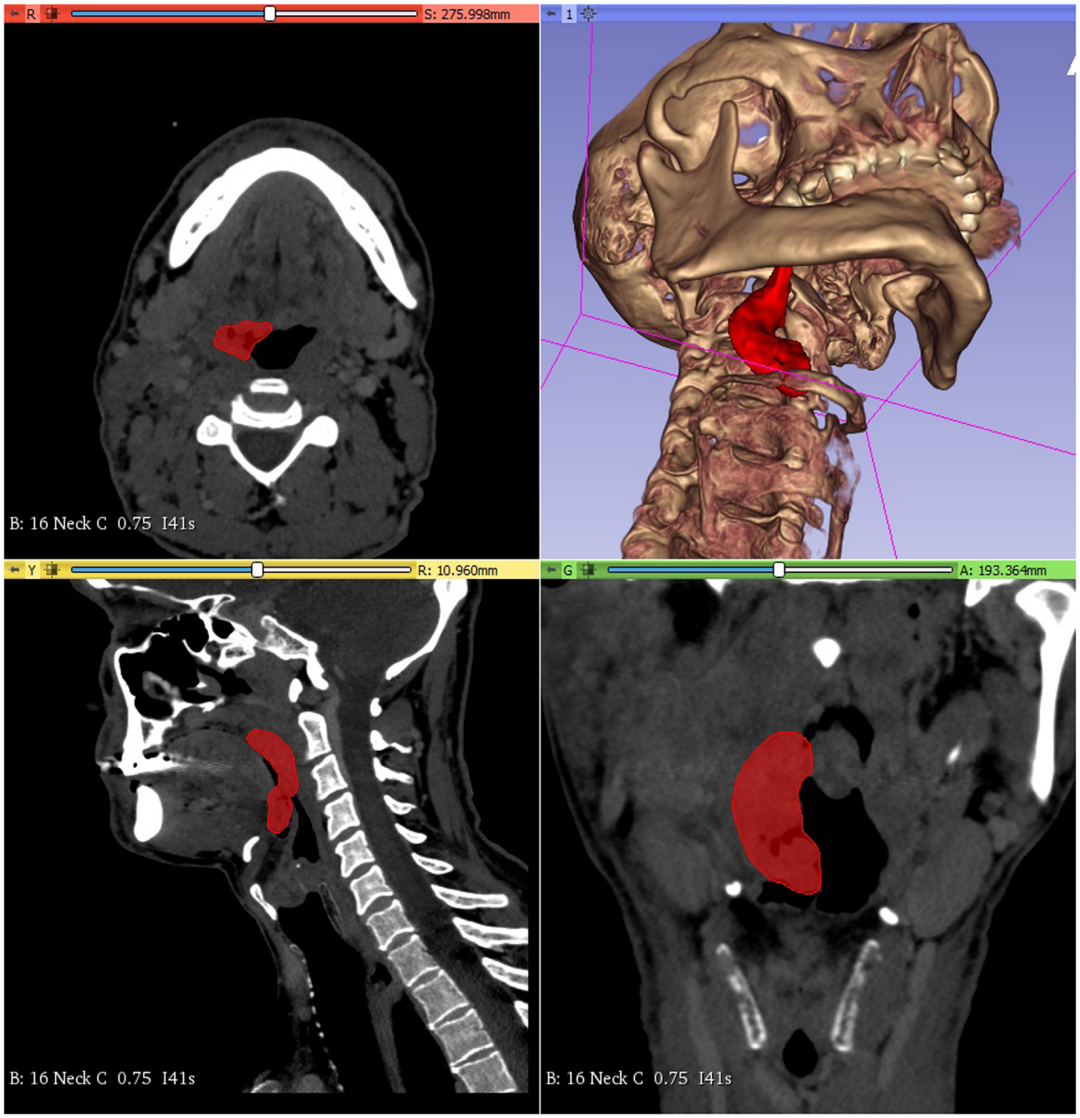
HR CT image and 3D model of a HPV negative stage T3 tonsillar squamous cell carcinoma. Tumor mass is marked red on the horizontal, sagittal and frontal planes in all sections and a 3D model is built up based on the CT segments on the upper right quarter of the picture.

**Figure 6:**
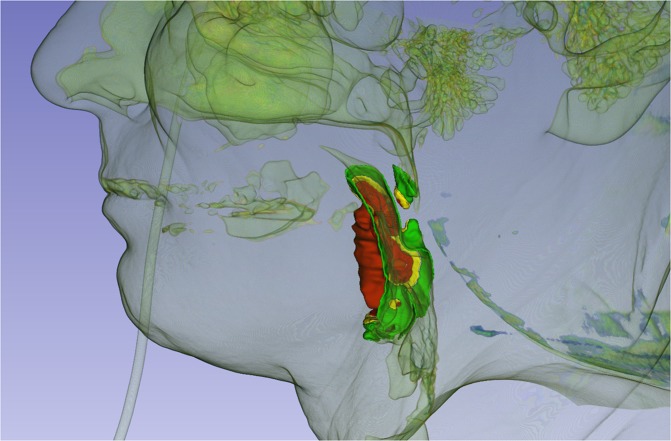
3D image of HPV negative stage T3 right side exulcerated tonsillar squamous cell cancer - with the anatomical extension into the vallecula and infiltrating the lateral pharyngeal wall (deep orange)- and peritumoral mucosa at 1 cm from the tumor margin (red tone), at 2 cm (yellow) and 3 cm (green) from the tumor margin in sagittal view.

**Figure 7:**
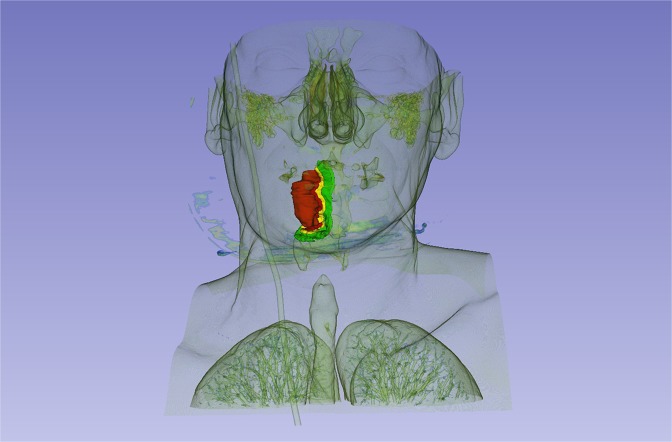
3D image of HPV negative stage T3 right side exulcerated tonsillar squamous cell cancer - with the anatomical extension into the vallecula and infiltrating the lateral pharyngeal wall (deep orange)- and peritumoral mucosa at 1 cm from the tumor margin (red tone), at 2 cm (yellow) and 3 cm (green) from the tumor margin in frontal view.

The patient’s HR CT DICOM files were uploaded to 3D slicer version 4.1. The 3D model was generated based on a HPV negative right side exulcerated tonsillar tumor (stage T3) with the anatomical extension into the vallecula and deeply infiltrating the lateral pharyngeal wall. The area pointed red on the high resolution CT shows the primary tumor. It is visualized by 3D slicer program in an anatomical model also appearing with deep orange color (Fig. 5). In this 3D model we were able to visualize the tumor propagation into the surrounding mucosal tissues where the biopsy samples were taken from and evaluated for miRNA expression levels. On the images the clinical tumor mass is painted deep orange, peritumoral mucosa at 1 cm is inked red, 2 cm is yellow and 3 cm is green (Figs. 6,7).

## Discussion

Growing interest has focused on the development of novel treatment strategies based on HNSCC cell biology. Within this trend, recent identification of human papilloma virus (HPV) positive HNSCC is a milestone discovery, because it is has become apparent that this type of cancer has a distinctive genetic and epigenetic profile, which can be cured within the framework of conventional organ-preserving treatment^29–33^.

HPV genotyping experiments, have demonstrated that HPV 16 has the highest percentage in oropharyngeal tumors and in a smaller proportion, the presence of HPV 33 and 35 genotypes have also been confirmed^34^. In our study we applied a genotyping platform which is able to recognize a broad spectrum of mucosal HPV genotypes.

We could confirm HPV 16 as the most frequent genotype in all our clinical samples (7 cases out of 48) and we also detected HPV 52 besides HPV 16 in a single case. HPV 52 belongs to the high cancer inducing risk category (HR) HPV group and it was found to be the third most prevalent genotype- after HPV 42 and HPV 16- during the routine cervical HPV genotyping at our laboratory site. In the head and neck region the oropharynx presents the highest ratio of HPV associated HNSCC. The mucosal surface of lacunas and crypts constitute a strong conjunction between the epithelial mucosa and the lymphatic tissue underneath and is often revealed in the background of tonsillar predilection of HPV initiated carcinogenesis. During our study period between 2017 and 2018, 48 patients were diagnosed with oropharynx, hypopharynx and larynx HNSCCs. From this patient cohort 25 patients had orophyaryngeal tumor and were selected into the study for HPV genotyping and microRNA expression characterization. Due to the very prospective manner of the study (the patients were diagnosed, than mapping biopsy was carried out and biopsies was analyzed for HPV with other genetic features of the samples) whilst we gained HPV negative and HPV positive case numbers according to the general HPV incidence in our region. Although the quantity of HPV positive samples used in our statistical analysis restrict the interpretation of our results due to its smaller number compared to the HPV negative tumors (8 HPV positive cases compared to 17 cases of HPV negatives in the T2-3 stage oropharyngeal HNSCCs). To fulfill the need for group equalizing we should have waited a two-year period to be able to complete sample size for HPV positive tumor group. Since our study is a proof of concept for the 3D model combined with miRNA expression analysis we presume that case number would not influence much on the outcome. Also it is hard to reflect the current results on HPV positivity of the HNSCCs found in our region, because it is known that geographic distribution and the prevalence of certain HPV genotypes may differ, which can cause differences in tumor induction.

The molecular pathways of viral and chemical carcinogenesis are different resulting in alterations in miRNA profiles^35^. In our study we found differences in miRNA expression patterns in the HPV positive and negative tumor tissues as well as in the surrounding mucosal fields.

We found miR-21-5p expression to reach higher levels in HPV positive tumors and 1 cm distant peritumoral area. In study evidences published so far, miR-21 showed significantly lower expression in HPV positive head and neck tumors, compared to HPV negative ones^36–38^. However, these studies lacked the clinical and histopathological homogeneity of their samples. Sample enrollment was heterogenic for the clinical stage. Furthermore, tumor negative samples were collected close to the tumor itself; consequently, these studies neglected to take in consideration the tumor organization field. Our study samples were all taken from homogeneous, advanced stage (T2, T3) tumor propagations for both HPV negative and positive tumors, so our results reflect miR-21-5p characteristics of clinicopathologically homogenous tumor groups. Our tumor negative samples were collected according to a complete mapping biopsy strategy taking into consideration the TOF (tissue organization field).

miR-143 is a tumor suppressor miRNA, which has been identified as having a lower expression level in HPV associated head and neck tumors. It inhibits the proteins E2F (E2 transcription factor) and survivin and their protein translation, which contributes to tumor proliferation and neoangiogenesis^39^. It is associated with a better prognosis, hence its higher expression was observed in HPV positive oropharyngeal tumors^40^. We have also found a high miR-143 expression in HPV positive tumors compared to the negatives, but the difference was more characteristic for the peritumoral mucosa, which showed significantly higher concentrations of miR-143.

As well as miR-21-5p, we recognized miR-221-5p with significantly higher expression levels in the HPV positive cancers compared to the negatives, which may also be explained by the fact that HPV positive tumors were large in size. On our mapping biopsy evaluation the highest miRNA values were measured for the HPV positive tumor tissues and miR-221-5p expression. In conclusion, our findings confirm that miR-221 is involved in HPV positive HNSCC tumor growth.

Regarding the HPV negative HNSCCs despite the exponentially expanding information on the cellular biology, research evidence has not lead to a holistic understanding of the dynamically evolving molecular circuitry of this type^33^. Many factors seem to contribute to their development, such as the accumulation of genetic and epigenetic alterations, e.g. chromosomal abnormalities, inactivation of tumor suppressors, and increased activity of oncogenes. Signaling pathways in antitumor mechanisms are involved in tumor initiation and progression correlating with biological aggressiveness: TP53 (tumor protein p53), RB1/INK4/ARF (retinoblastoma-associated protein 1/infestin 4/alternate open reading frame) and NOTCH1 (neurogenic locus homolog protein 1) signaling pathways are recognized. Data from DNA sequence analysis implies that the phosphatidylinositol 3-kinase PI3K-Akt-mTOR pathway is also frequently involved in the cancer development^41^.

Recent rapid progress in epigenetic approach has revealed that noncoding RNAs (ncRNA), especially miRNA, have a fine-tuner role within gene transcription. Through interaction with mRNA and chromatin modulators, miRNA orchestrates the stoichiometric equilibrium of gene expression on dynamically changing cellular context^33^. Thereby, miRNA behaves as a morphostatic factor, linking epigenetic to genetic factors, it is characterized by distinct genetic attributes and plays a key role in the regulation of the mucosal landscape and has been proven to be useful in the molecular characterization of several tumor types^41^. In our study, we utilized the benefits of this molecule to detect and characterize the HNSCC tissue organization field. Considering the quantitative differences in the miRNA expression, we found many alterations between tumors and phenotypically intact tissues at defined distances. We revealed significant changes of miR-21-5p and miR-221-5p in HPV negative tumors. Tumor surrounding normal mucosa showed gradual concentration depletion of these markers.

In previous studies, the role of miR-21 has been demonstrated in the inhibition of protein translation of the mRNA of the p53 gene responsible for cell cycle regulation in malignant melanoma and oral squamous cell carcinoma^42^. An elevated expression of miR-21 was also reported in squamous cell carcinomas of the head and neck^43,44^, including larynx^45^, pharynx and tongue tumors, primarily metastatic squamous cell carcinomas of the tongue in the latter^46^. The increased expression of miR-21 is closely related to a low five-year survival rate and lower response to chemoradiotherapy, which can be an important tool for treatment planning and prognosis estimation in the future^36,37^. Currently, there is no evidence in relation to mapping biopsy in literature, but during the progress of our examinations, we found high values of miR-21-5p in the healthy tissue at a distance of 1 cm from the tumor, which correlates with the surgical safety zone.

miR-155 is a type of miRNA, identified in squamous cell cancers of the larynx, it promotes tumor invasion by blocking the SOC (suppressor of cytokine signaling proteins) and STAT (signal transducer and activator of transcription) transcription factors^47^. It can be closely associated with a local recurrence in the case of oropharyngeal cancer^48^, and its expression is higher in laryngeal tumors^49^. It also correlates with tumor proliferation, tendency for metastasis and poor prognosis^50–53^. The changes in biomarker values in miR-155, measured at different biopsy distances, could not prove the role of miR-155 in the peritumoral field in our study.

A previous study by *Hussein et al*. confirmed decreased miR-143 expression within the HNSCC tumor tissue, compared to the values measured in the mucosal controls^54,55^. In our study, we found a higher expression in tumor samples than in normal tissue, however the tissues taken at 1 cm distance in the intact mucosa showed similar, and in some cases higher expression levels compared to the tumor samples themselves. Changes were found to be very moderate. The expression results decreased with the distance in the peritumoral field.

High expression of miR-221 has been demonstrated by several previous studies in the case of laryngeal squamous cell carcinoma^56^ and head and neck squamous cell carcinoma^57^, while low expression has been reported in tumor-free tissue. We can confirm these results for miR-221-5p as our tissue organization field model, showed a significant gradual change on the tumor surrounding mucosal surface.

Overall, it can be said that the differing patterns of HPV positive tumors can explain the prognostic and behavioral differences compared to HPV negative tumors. In order to perform a comprehensive examination regarding epigenetic differences, additional investigations are needed with more cases involved. We plan to examine regulation of the miRNAs, we have analyzed thus far, through their target mRNAs in a future research project.

In the CT based 3 D model we tried to visualize the tumor mass and the peritumoral tissues considering our mapping biopsy strategy and the tissue miRNA expression results. In the 3D model the clinical tumor mass and the peritumoral tissue locating at 1 cm distance from the macroscopically tumorous margin - where similar miRNA patterns were proved - presents in red tone colors with precise compilation (Figs. 6,7). Surgical excision carried out in this zone can result a high risk of tumor recurrence. Yellow color represents peritumoral tissue at 2 cm distance from the tumor margin. This is a safety field with a better epigenetic patterns differing from the tumor. In case of resecable tumors the surgical resection is highly recommended in this area to avoid the locoregional recurrence. This model can be utilized in preoperative surgical planning to decide the optimal tumor resection line. In case the surgical line affects the functionality (swallowing, breathing and speaking) we prefer the organ preservation among therapeutical modalities (chemoirradiation). The 3D software might be effective in assisting preoperative planning, especially in current clinical settings. Our approach enables the risk estimation of malignant disease progression before the operative intervention on the basis of genomic and epigenetic characteristics of the tumor and the peritumoral mucosal surface and the surgical safety margin can be determined in consideration with the anatomical situation of the mucosal landscape. This strategy could facilitate the complex planning of surgical excision. In some cases, where the anatomy does not permit a more extended surgery according to the 3D model and tumor removal was carried out in the 1 cm zone which is still histologically tumor-free but high recurrence risk is indicated by genomic evaluation a closer patient follow-up is highly recommended.

HPV positive cases, where organ preservation and chemo-irradiation therapy are usually preferred, could also be managed in a more precise way based on the 3D models and genomic cancer field evaluation.

Our ultimate goal was to optimize the diagnostic steps in HNSCC management, to estimate and minimalize the chances of tumor recurrence and to achieve better survival with higher life quality of HNSCC patients.

### Ethics approval and consent to participate

The clinical and experimental study protocol described here was performed under the license number 2014/5341 (University of Pécs, regional ethical committee). The methods were carried out in accordance with the relevant guidelines and regulations. Informed consent was obtained from all participants who were enrolled in this study.

## Supplementary information

Supplementary Information

## Data Availability

All data generated during this study are included in this published article.
