# Author Correction: Visualization of mucosal field in HPV positive and negative oropharyngeal squamous cell carcinomas: combined genomic and radiology based 3D model

**DOI:** 10.1038/s41598-020-62491-0

**Published:** 2020-03-24

**Authors:** Eva Orosz, Katalin Gombos, Nerina Petrevszky, David Csonka, Istvan Haber, Balint Kaszas, Arnold Toth, Krisztian Molnar, Krisztina Kalacs, Zalan Piski, Imre Gerlinger, Andras Burian, Szabolcs Bellyei, Istvan Szanyi

**Affiliations:** 1 University of Pécs, Medical School, Department of Otorhinolaryngology, Pécs, Hungary; 2 University of Pécs, Medical School, Department of Laboratory Medicine, Pécs, Hungary; 3 University of Pécs, Faculty of Engineering and Information Technology, Pécs, Hungary; 4 University of Pécs, Medical School, Department of Pathology, Pécs, Hungary; 5 University of Pécs, Medical School, Department of Radiology, Pécs, Hungary; 6 University of Pécs, Medical School, Department of Oncotherapy, Pécs, Hungary

Correction to: *Scientific Reports* 10.1038/s41598-019-56429-4, published online 08 January 2020

In this Article, the legend of Figure 1 is incorrect:

“Squamous cell epithelium negative for p16 immunohystochemical staining at 40x magnification. Histological section originates from a grade 2 oropharyngeal wall squamous cell carcinoma.”

should read:

“Squamous cell epithelium positive for p16 immunohistochemical staining at 40x magnification. Histological section originates from a grade 3 tonsillar squamous cell carcinoma.”

In addition, the legend of Figure 2 is incorrect:

“Squamous cell epithelium positive for p16 immunohystochemical staining at 40x magnification. Histological section originates from a grade 3 tonsillar squamous cell carcinoma.”

should read:

“Squamous cell epithelium negative for p16 immunohistochemical staining at 40x magnification. Histological section originates from a grade 2 oropharyngeal wall squamous cell carcinoma.”

