# Supplementary figures and images for: Visualization of mucosal field in HPV positive and negative oropharyngeal squamous cell carcinomas: combined genomic and radiology based 3D model
Source: Sci Rep. 2020 Jan 8;10:40. doi: 10.1038/s41598-019-56429-4 (PMC6949264; doi:10.1038/s41598-019-56429-4)

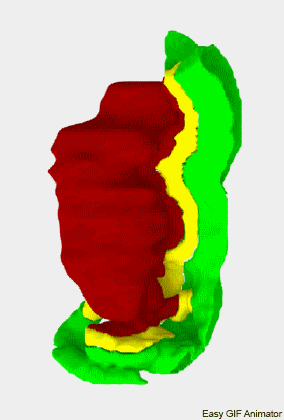

Supplement: Supplementary file 1 — Supplementary Information [file 41598_2019_56429_MOESM1_ESM.gif]
